# Thyroid-like follicular carcinoma of the kidney: A report of two cases and literature review

**DOI:** 10.3892/ol.2014.2027

**Published:** 2014-04-03

**Authors:** YUN-ZHI LIN, YONG WEI, NING XU, XIAO-DONG LI, XUE-YI XUE, QING-SHUI ZHENG, TAO JIANG, JIN-BEI HUANG

**Affiliations:** Department of Urology, The First Affiliated Hospital of Fujian Medical University, Fuzhou, Fujian 350005, P.R. China

**Keywords:** thyroid-like follicular carcinoma of the kidney, features, treatment, prognosis

## Abstract

There have only been a few reports of thyroid-like follicular carcinoma of the kidney (TLFCK) to date. In the present study, two patients with TLFCK are reported. Patient 1 was a 65-year-old male exhibiting repeated hematuria and right back pain. No tumors were located in the patient’s thyroid or lungs. The physical examination revealed percussion tenderness over the right kidney region was noticed. Enhanced computed tomography (CT) indicated a right renal pelvic carcinoma, for which the patient underwent a radical right nephrectomy. Patient 2 was a 59-year-old male with a mass in the right kidney, located during a health examination and who exhibited no obvious clinical symptoms. The patient was clinically diagnosed with right renal carcinoma, confirmed by an enhanced CT. The patient underwent a radical right nephrectomy. The clinical features, imaging results, pathology, immune phenotypes, treatment and prognosis were analyzed. The associated literature was also reviewed. The cut surface of each tumor showed gray-white material with a central solid area, including scattered gray-brown necrotic and gray hemorrhagic areas and small cystic cavities. Microscopically, the arrangement of the tumor cells mimicked thyroid follicles with red-stained colloid-like material in the lumen. No renal hilar lymph node involvement was noted. The tumor tissue of patient 1 was immunohistochemically positive for vimentin, epithelial membrane antigen (EMA), cytokeratin (CK), CK7, and neuron specific enolase; and negative for CK34BE12, synapsin (Syn), CK20, cluster of differentiation 56 (CD56), CD10, Wilm’s tumor-1 (WT-1), CD34, CD57, P53, CD99, thyroid transcription factor-1 (TTF-1), CD15 and thyroglobulin (TG); with a Ki-67 labeling index (LI) of 30%. The tumor tissue of patient 2 was immunohistochemically positive for vimentin, EMA, CK7 and CK20; and negative for CD56, CD10, WT-1, CD34, CD57, P53, CD117, TTF-1, CD15, CD99, TG, chromogranin A and Syn; with a Ki-67 LI of 20%. TLFCK is a rare renal tumor with low malignancy but medium invasiveness. It morphologically resembles thyroid follicular carcinoma but does not express TTF-1 or TG. Radical nephrectomy can achieve good patient outcomes.

## Introduction

Thyroid-like follicular carcinoma of the kidney (TLFCK) is microscopically similar to thyroid follicular carcinoma (1). TLFCK is a rare pathological type of renal tumor, with potential origins in the kidney cells. This emerging entity has not been included in the World Health Organization Classification of Tumors: Pathology and Genetics of Tumors of the Urinary System and Male Genital Organs, and its biological behavior has not yet been determined (2). TLFCK was documneted by Jung *et al* for the first time in 2006 (1). To date there have only been 13 complete case reports and clinical physician’s understanding of its clinical features and pathological characteristics remains inadequate. The existing cases of TLFCK normally occur in young and middle-aged females (eight of 13 cases). The majority of the patients are without obvious clinical symptoms, certain patients present with hematuresis or waist pain. All patients were treated with surgery. Radical nephrectomy is capable of achieving successful patient outcomes. In the present study, two patients with TLFCK are reported who were treated at The First Affiliated Hospital of Fujian Medical University (Fushou, Fujian, China) between 2011 and 2013. The clinical manifestations, diagnosis, pathology and treatment of these patients and other patients described in the literature are discussed. Written informed consent was obtained from the patients.

## Case report

### Patient 1

**Figure 1:**
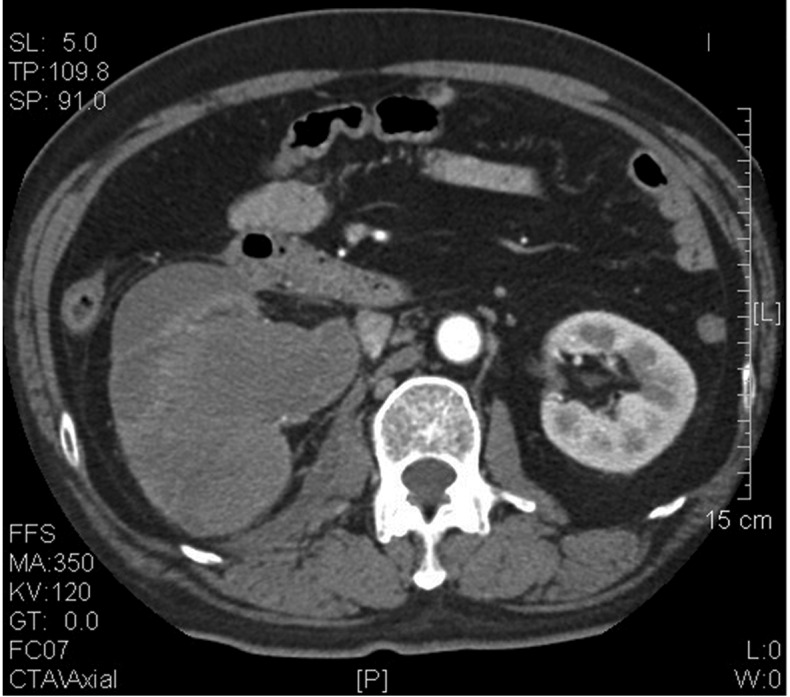
Pre-operative computed tomography examination of patient 1, showing a mass in the right renal pelvis, indicating a right renal pelvic carcinoma.

A 65-year-old male was admitted for repeated hematuria during urination for four years and right back pain for seven days. The patient had no tumor family history. The physical examination upon admission showed normal vital signs, and normal findings of the heart, lungs, liver and spleen. Percussion tenderness over the right kidney region was noticed. Plain magnetic resonance imaging revealed a mass in the right kidney, which was possibly a renal carcinoma with involvement of the renal fascia. Color Doppler ultrasonography confirmed the presence of a solid mass with hypoechogenicity in the right renal hilum. Enhanced computed tomography (CT) indicated a right renal pelvic carcinoma (Fig. 1), for which the patient underwent a radical right nephrectomy on January 14, 2011. Intraoperatively, the tumor measured 8.0×4.3×5.0 cm and had a hard texture. Gerota’s fascia and lymph nodes that are adjacent to the kidney and abdominal aorta were not involved. A postoperative test showed no signs of hyperthyroidism. The patient had no personal or family history of hyperthyroidism. Ultrasonography found no tumor or other abnormal signs in the thyroid and other body parts. Routine blood tests and thyroid-stimulating hormone (TSH), triiodothyronine (T3) and thyroxine (T4) concentrations were within normal limits. Following the surgery, the patient has shown no signs of tumor recurrence or metastasis.

**Figure 2:**
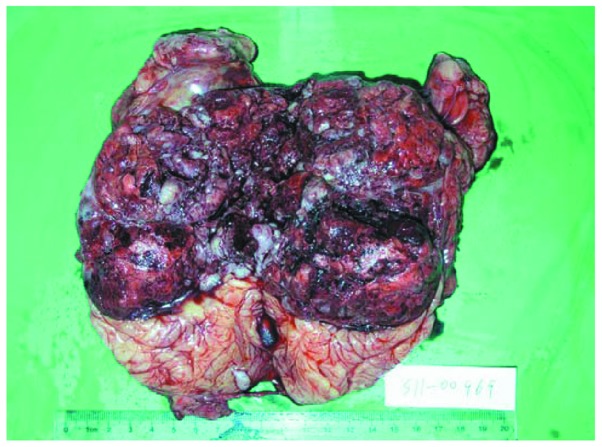
Postoperative results of patient 1: The resected kidney was 13.0×7.0×6.0 cm and the tumor was 8.0×4.3×5.0 cm in size. The cut surface revealed a well-circumscribed gray-white solid tumor, and the gray-red hemorrhagic areas are observed with small cystic cavities.

**Figure 3:**
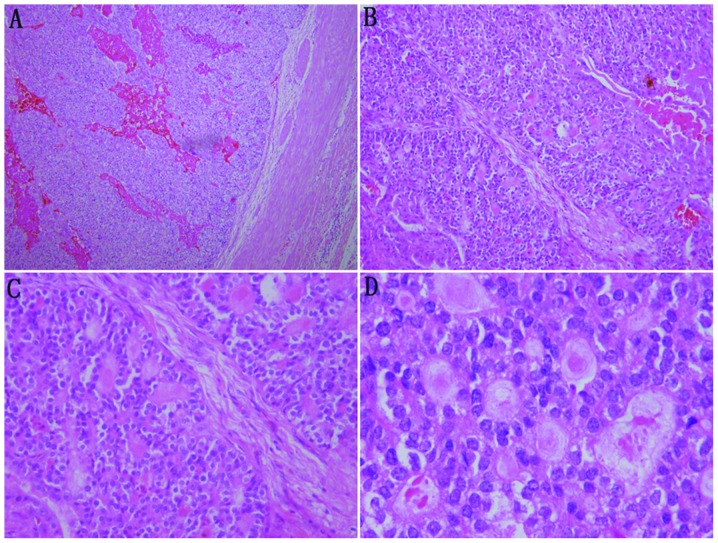
Microscopic examination of the tumor in patient 1. (A) The tumor is encapsulated with a fibrous pseudo-capsule (×40). (B) The tumor cells are separated by mesenchymal tissues into patches with a morphology of ‘dried follicles’ (×100). (C) The tumor cells show a morphology mimicking thyroid follicles with red-stained colloid-like material in the lumen. The colloid-like material broke a number of the lumens and merged (×200). (D) The nuclei are enlarged and overlapped in round, oval or spindle shapes with fine chromatin pattern and one to two inconspicuous nucleoli per nucleus (×400). Hematoylin and eosin staining.

Macroscopically, the resected kidney measured 13.0×7.0×6.0 cm, with the tumor located in the renal parenchyma measuring 8.0×4.3×5.0 cm. The cut surface revealed a well-circumscribed gray-yellow solid tumor. Scattered gray-yellow necrotic areas and gray-red hemorrhagic areas were observed with small cystic cavities (Fig. 2). Microscopically, the tumor cells showed a morphology similar to thyroid follicles or a sieve, occupying over 50% of the visual field. The follicular lumens were filled with evenly red-stained colloid-like material. Vacuoles were rarely observed around the lumens. The colloid-like material broke a number of the lumens and merged, resulting in the tumor cells exhibiting a morphology of ‘dried follicles’. The tumor cells showed indistinctive nuclear heteromorphism, reduced transparent cytoplasm and unclear borders. No clear cell type or other type of renal cell carcinoma was identified (Fig. 3).

**Figure 4:**
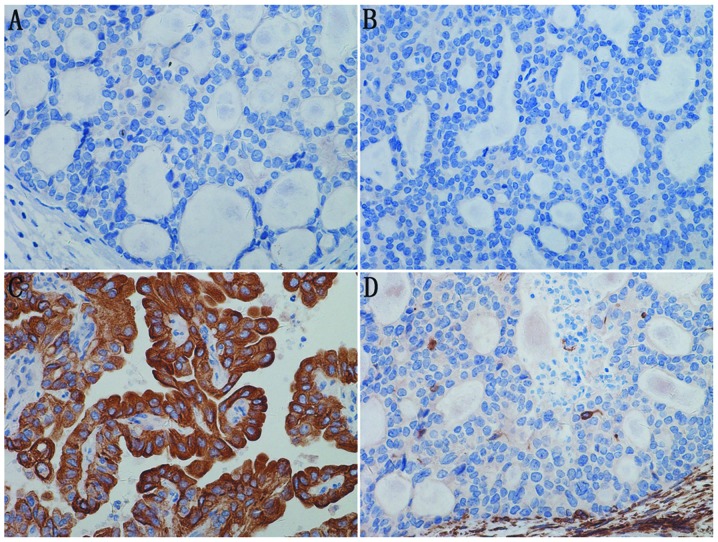
Immunohistochemistry of the TLFCK in patient 1 (×400). (A) TG(−), (B) TTF-1(−), (C) CK(+) and (D) Vimentin(+). TLFCK, thyroid-like follicular carcinoma of the kidney; TG, thyroglobulin; TTF-1, thyroid transcription factor-1; CK, cytokeratin. EnVision staining.

Immunohistochemically, the tumor was positive for vimentin, epithelial membrane antigen (EMA), cytokeratin (CK), CK7 and neuron specific enolase (NSE); and negative for CK34BE12, synapsin (Syn), CK20, cluster of differentiation 56 (CD56), CD10, Wilm’s tumor-1 (WT-1), CD34, CD57, P53, CD99, thyroid transcription factor-1 (TTF-1), CD15 and thyroglobulin (TG); and had a Ki-67 labeling index (LI) of 30%. The pathological diagnosis was TLFCK (Fig. 4).

### Patient 2

**Figure 5:**
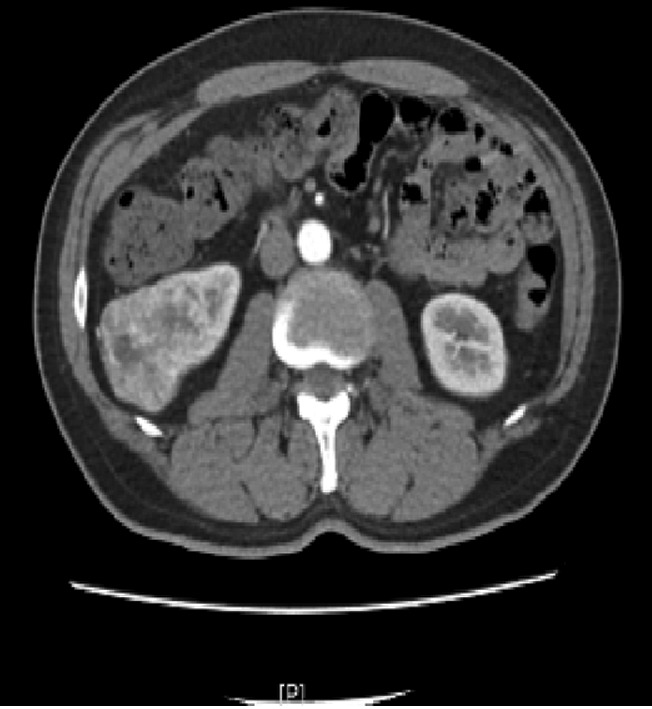
Pre-operative computed tomography image of patient 2, showing a mass in the middle-lower pole of the right kidney, extending out of the profile of the kidney. The renal fascia adjacent to the mass is thickened indicating a renal carcinoma.

A 59-year-old man was found to have a mass in the right kidney during a routine health examination and was admitted. The patient had no personal or family history of thyroid dysfunction. A physical examination showed normal vital signs, heart, lungs, liver and spleen, and no percussion tenderness over the right kidney region. Color Doppler ultrasonography revealed a mass in the right kidney, which was confirmed by enhanced CT (Fig. 5). Owing to the possibility of malignancy, the patient was initially diagnosed with renal carcinoma, for which radical right nephrectomy was performed on May 3, 2013. During the surgery, the tumor was found to be located in the middle-lower pole of the right kidney. It measured 6.0×4.5×5.0 cm and had a hard texture. The tumor was well-circumscribed with a mild adhesion to the adjacent tissues. Gerota’s fascia and the lymph nodes that are adjacent to the kidney and abdominal aorta were not involved. Ultrasonography found no tumor or other abnormal signs in the thyroid and other body parts. Routine blood tests, TSH, T3 and T4 were within normal limits. Following the surgery there has been no evidence of tumor recurrence or metastasis.

**Figure 6:**
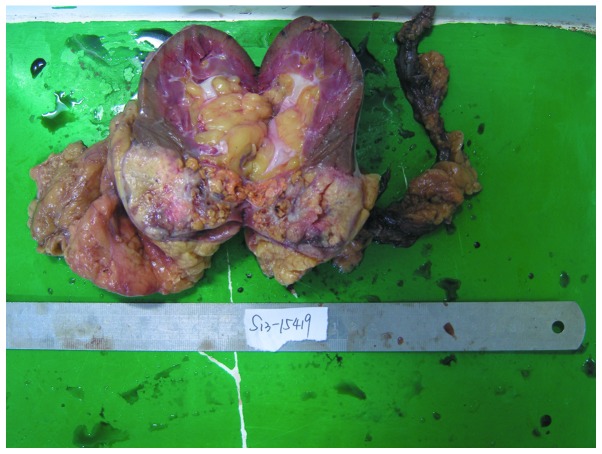
Postoperative results in patient 2. The resected kidney measured 14.0×7.0×7.0 cm and the tumor was 6.0×5.0×5.4 cm. The cut surface revealed a solid, gray-white, well-circumscribed tumor with gray-yellow ischemic areas and small cystic cavities.

The resected kidney measured 14.0×7.0×7.0 cm, with the tumor measuring 6.0×5.0×5.4 cm, located in the middle-lower pole. The cut surface revealed a solid, gray-white, well-circumscribed tumor with invasion of the surrounding renal tissues and capsules. Scattered gray-yellow ischemic areas and small cystic cavities were also observed (Fig. 6).

**Figure 7:**
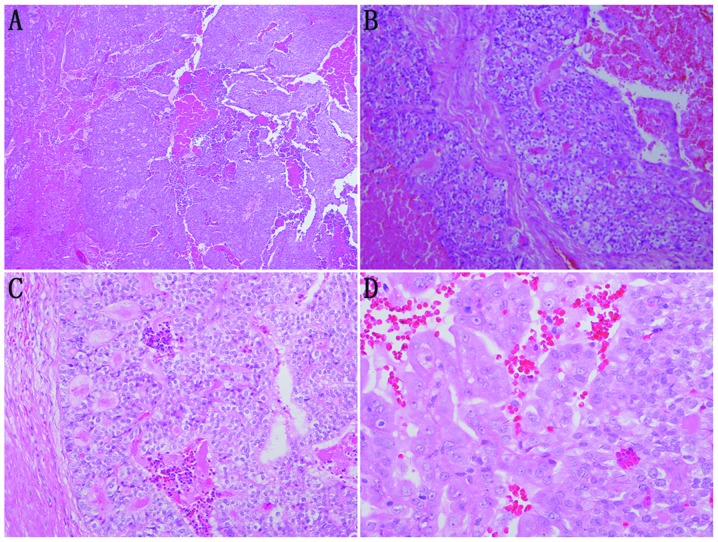
Microscopic examination of the tumor in patient 2. (A) The tumor cells are separated by mesenchymal tissues into patches with a morphology of ‘dried follicles’ (×100). (B) The tumor cells show a morphology mimicking thyroid follicles or a sieve with red-stained colloid-like material in the lumen (×100). (C) The colloid-like material broke and a number of the lumens and merged. The tumor was covered with a thin fibrous pseudo-capsule (×200). (D) The nuclei are mostly eosinophilic, enlarged and overlapped in round, oval or spindle shapes with fine chromatin pattern and one to two inconspicuous nucleoli per nucleus (×400). Hematoylin and eosin staining.

Microscopically, the tumor was covered by a fibrous pseudocapsule. The majority of the tumor cells were arranged as thyroid follicles or a sieve, with certain cells appearing in patches or fibrous septae. The follicular lumens were filled with evenly red-stained colloid-like material. Vacuoles were rarely observed around the lumens. The colloid-like material broke a few of the lumens and merged, resulting in the tumor cells exhibiting a morphology of ‘dried follicles’. The tumor cells showed a reduced transparent cytoplasm and unclear borders. The nuclei were enlarged and overlapped in round, oval or spindle shapes with a fine chromatin pattern and one to two inconspicuous nucleoli per nucleus. The nuclear groove was unremarkable and mitosis was rarely observed. No clear cell type or other type of renal cell carcinoma was identified (Fig. 7).

**Figure 8:**
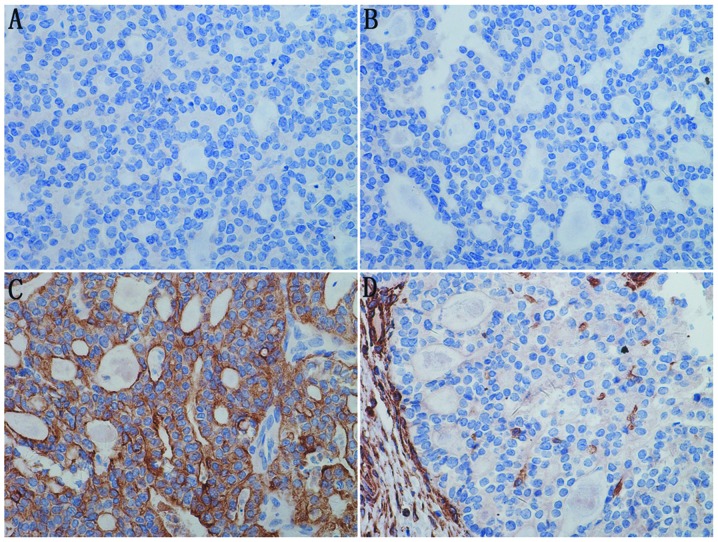
Immunohistochemistry of the TLFCK in patient 2 (×400). (A) TG(−), (B) TTF-1(−), (C) CK(+) and (D) Vimentin(+). TLFCK, thyroid-like follicular carcinoma of the kidney; TG, thyroglobulin; TTF-1, thyroid transcription factor-1; CK, cytokeratin. EnVision staining.

Immunohistochemically, the tumor cells were positive for vimentin, EMA, CK7 and CK20; and negative for CD56, CD10, WT-1, CD34, CD57, P53, CD117, TTF-1, CD15, CD99, TG, chromogranin A (CgA) and Syn; and had a Ki-67 LI of 20% (Fig. 8).

## Discussion

The first case of TLFCK was described in 2006; all cases of TLFCK reported are summarized in Table I. The locations and clinical manifestations of TLFCK do not distinguish these tumors from other types of renal carcinoma. The majority of patients with TLFCK are adults, aged 22 to 83 years, with a female to male predominance (8:5, including the two patients in the present study) (1–5). The majority of tumors involve the right kidney more than the left, as is the case in the present study.

The tumors in the present study were pathologically confirmed as having originated in the renal parenchyma, with well-circumscribed pseudo-capsules. The cut surfaces of the tumor were yellow-white or gray-white with focal necrosis. There was no evidence of morphology that is typical of clear cell type renal carcinoma, consistent with previous reports (2–4). The sizes of TLFCK have been reported to range from 1.9–11.8 cm (1–2). The most significant microscopic feature of TLFCK is the striking resemblance to the well-differentiated follicular carcinoma of the thyroid gland, with follicular structures and colloid-like material. By contrast, no TLFCK has shown morphological features similar to clear or other types of renal carcinoma (2–4).

The tumors in the present study were negative for TTF-1 and TG, ruling out the possibility of metastasis from thyroid tumors and supporting a diagnosis of TLFCK (1–5).

WT-1 expression was found to be immunohistochemically positive in TLFCK tumor cell nuclei, indicating that these tumors originate in the kidneys (3). Thyroid follicle-like structures have been observed in patients with chronic pyelonephritis and end-stage renal disease, indicating that TLFCKs may originate from renal tubular epithelial cells (2).

The immune phenotypes of reported TLFCK are listed in Table II. Immunostaining results for epithelial markers have differed among studies; therefore, a combination of epithelial cell and renal tubular epithelial cell markers have been used in the majority of studies (2–4). Assays of primary and metastasized tumors of TLFCK have found that paired box gene 2 (PAX2) and PAX8 are expressed, whereas TG and TTF-1 are not, supporting the renal origin of this malignancy (6).

Chromosomal analysis has shown gains of chromosomes 7q36, 8q24, 12, 16, 17p11-q11, 17q24, 19q, 20q13, 21q22.3 and Xp (1), and losses of chromosomes 1p36, 3 and 9q21–33 (1) and 1, 3, 7, 9p21, 12, 17 and X (5) in TLFCK. TLFCKs were shown to be positive for the expression of 135 genes but negative for an additional 46 genes (2). A 2.5-fold increase in the expression of mixed lineage leukemia gene has been observed; this gene encodes a transcription factor that is involved in the development of several types of hematological malignancies, such as acute leukemia (7).

TLFCK should be differentiated from renal metastases from thyroid follicle carcinoma, however, only 10 cases of the latter have been reported (8,9). Thyroid follicle carcinomas tend to metastasize to lymph nodes, lung and bone. Renal metastasis of thyroid follicle carcinoma usually occurs following multiple systemic metastases. The majority of thyroid carcinomas are markedly positive for TTF-1 and TG, although these two proteins may not be expressed by certain poorly differentiated or sarcoma-like thyroid carcinomas (10). Therefore, further examination is required to diagnose TLFCK. TLFCK should also be differentiated from malignant ovarian teratoma with thyroid tissue as the sole component. Renal metastasis from these malignancies can be ruled out by imaging of the ovaries (11,12). In addition, the absence of expression of TTF-1 and TG can be diagnostic of TLFCK, and TLFCK should be differentiated from other renal carcinomas. Rare renal carcinoid tumors can have a follicular structure and red-stained colloid material, but these tumors have smaller nuclei, finer chromatin and no evidence of necrosis. Neuroendocrine carcinomas are characterized by nuclear heteromorphism and frequent mitosis, and a clear positivity for NSE, CD56, CgA and synaptophysin. Epithelial-type nephroblastomas usually occur in children with undifferentiated embryo, epithelial and mesenchymal tissues. This malignancy usually shows epithelial rosettes, but no follicular structure or colloid-like material has been reported.

Radical nephrectomy is the major treatment method for TLFCK and can achieve good prognosis (1,2,6). Patient 1 has remained disease-free for over two years, whereas patient 2 has shown no evidence of tumor relapse one month following the surgery. Follow-up of six TLFCK patients for a mean of 47.3 months identified that five were disease-free and one showed metastasis to renal hilar lymph nodes, indicating that these tumors have a low malignancy (2). One patient was found to develop metastasis to the left lower lung two months following surgery (5); the metastasis was surgically removed and the patient has shown no evidence of recurrence or metastasis during a follow-up period of five years. TLFCK is regarded as having medium invasiveness, but long-term survival can be achieved by radical resection of the tumor (6).

## Figures and Tables

**Table I:** Clinicopathological features of primary TLFCK reported between 2006 and 2012.

First author/s (ref)	Age, year	Gender	Presentation	Tumor location	Tumor size, cm (location)	TNM stage	Treatment	Disease-free survival
Amin *et al* (2)	53	Female	Incidental	Right kidney (middle pole)	2.1	pT1aNx	RN	4 years and 6 months
	29	Female	Incidental	Right kidney (upper pole)	1.9	pT1aNx	RN	7 years
	45	Male	Incidental	Right kidney (lower pole)	3.5	pT1aN1	RN	1 year and 5 months
	83	Male	Incidental	Left kidney (lower pole)	2.1	pT1aNx	RN	4 years
	35	Male	Incidental	Right kidney (middle pole)	3.0	pT1aNx	RN	1 year and 8 months
	50	Female	Incidental	Right kidney (middle pole)	4.0	pT1aN0	RN	7 months
Jung *et al* (1)	32	Female	Incidental	Right kidney (middle and lower poles)	11.8	pT2Nx	RN	6 months
Xu and Zang(3)	36	Female	Hematuria of the middle course of urination with blood clots	Left kidney (middle-lower pole)	10.0	pT2Nx	RN	1 year
He *et al* (4)	22	Female	Painless hematuria	Left kidney	8.0	pT1aN0	RN	No data
Sterlacci *et al* (5)	29	Female	Incidental	Left kidney (middle pole)	4.3	/	RN	Left lung lower lobe metastasis at 2 months, disease-free survival for 5 years
Dhillon *et al* (6)	34	Female	Hematuria and right back pain	Right kidney (middle pole) and bilateral lungs	6.3 (right kidney) 2.1 (left lung) 3.4 (right lung)	pT2N0M1	RN	1 year
Present study	65	Male	Hematuria and right back pain	Right kidney (middle-lower pole)	8.0	pT2N0	RN	15 months
	59	Male	Incidental	Right kidney (middle-lower pole)	5.2	pT1aN0	RN	1 month

RN, radical nephrectomy; TLFCK, thyroid-like follicular carcinoma of the kidney; TNM, tumor-node-metastasis.

**Table II:** Immune phenotypes of primary TLFCK.

First author/s (ref)	CK7	CK19	CK20	CK10	EMA	LCK	HCK	PCK	TTF-1	TG	PAX2	PAX8
Sterlacci *et al* (5)	+	NA	+	−	NA	NA	NA	NA	−	−	NA	NA
He *et al* (4)	Weak^+^	+	Weak^+^	Weak^+^	Weak^+^	NA	NA	+	−	−	NA	NA
Xu and Zang (3)	+	NA	NA	Focal^+^	+	NA	NA	NA	−	−	NA	NA
Dhillon *et al* (6)	+	NA	+	+	+	NA	NA	NA	−	−	+	+
Case 1	Focal^+^	+	−	−	+	NA	NA	NA	−	−	NA	NA
Case 2	+	NA	+	NA	+	NA	NA	NA	−	−	NA	NA

CK, cytokeratin; EMA, epithelial membrane antigen; LCK,; HCK,; PCK,; TTF-1, thyroid transcription factor-1; TG, thyroglobulin; PAX, paired box gene; TLFCK, thyroid-like follicular carcinoma of the kidney; LCK, lymphocyte-specific protein tyrosine kinase; HCK, hemopoietic cell kinase; PCK, pancytokeratin.
